# Kolaviron Improved Resistance to Oxidative Stress and Inflammation in the Blood (Erythrocyte, Serum, and Plasma) of Streptozotocin-Induced Diabetic Rats

**DOI:** 10.1155/2014/921080

**Published:** 2014-03-24

**Authors:** Omolola R. Ayepola, Nicole L. Brooks, Oluwafemi O. Oguntibeju

**Affiliations:** ^1^Nutrition and Chronic Disease Research Unit, Oxidative Stress Research Centre, Department of Biomedical Sciences, Faculty of Health and Wellness Sciences, Cape Peninsula University of Technology, Bellville, South Africa; ^2^Department of Wellness Sciences, Faculty of Health and Wellness Sciences, Cape Peninsula University of Technology, Cape Town, South Africa

## Abstract

*Aims.* Bitter kola seed (*Garcinia kola*, family: *Guttiferae*) has been used as a social masticatory agent in Africa for several years and is believed to possess many useful medicinal properties. The present study evaluates the antioxidative, anti-inflammatory, and antilipidemic effects of kolaviron (an extract from the *Garcinia kola* seeds) in the blood of streptozotocin- (STZ) induced diabetic rats. *Methods.* Diabetic rats were treated with kolaviron (100 mg/kg b*·*wt) orally, five times a week for a period of six weeks. Serum glucose and HBA_1C_ concentrations were estimated in experimental groups. The activities of antioxidant enzymes: catalase (CAT), superoxide dismutase (SOD), and glutathione peroxidase (GPX) (in erythrocytes) as well as plasma concentration of malondialdehyde (MDA), a product of lipid peroxidation, oxygen radical absorbing capacity (ORAC) and ferric-reducing antioxidant power (FRAP) were investigated. Serum levels of proinflammatory cytokines and growth factor: interleukin- (IL-) 1, monocyte chemotactic protein-1 (MCP-1), and vascular endothelial growth factor (VEGF), respectively, were also analyzed. *Results.* Kolaviron treatment markedly improved antioxidant status and abated inflammatory response evidenced by reduction in the levels of proinflammatory cytokines and growth factor, lipid peroxidation product, and the restoration of activities of erythrocyte antioxidant enzymes in the blood of diabetic rats. *Conclusion.* Kolaviron improved antioxidant status, reduced inflammation, and protected against hyperglycemic-induced oxidative damage in the blood of diabetic rats.

## 1. Introduction

Diabetes mellitus (DM) reduces life expectancy and adversely affects the quality of life of diabetic patients. According to the international diabetes federation (IDF), diabetic patients would increase from 371 million (2012) to 552 million in 2030. The limitations of the currently used antidiabetic drugs suggest an urgent need to discover new compounds that can serve as alternative and/or complementary therapy against this disease [1].

Hyperglycemia has been associated with an increased state of oxidative stress which is believed to play a crucial role in the onset and progression of late-diabetic complications through activation of stress-sensitive intracellular signaling pathways and the formation of gene products that causes cellular damage [2–4]. Biological free radicals are products of normal cellular metabolism and are maintained at a steady state level by antioxidants which act as free radical scavengers. At high concentrations, the production of free radicals overwhelms the detoxification capacity of cellular antioxidant system, resulting in oxidative stress and damage to cell structures [5, 6]. Altered antioxidant enzyme activities have been reported in the blood of diabetic patients and in diabetic animal models. Red blood cells (RBCs) are the first cells in the body to be exposed to stressful stimuli and hence prone to oxidative stress [7]. Damage to red blood cells by reactive oxygen species (ROS) results in abnormalities in the function, morphology, and metabolism of erythrocyte [8, 9]. Hyperglycemia and oxidation of membrane proteins are strongly associated with an increase in RBCs haemolysis and many pathological consequences [10]. Some of the mechanisms by which hyperglycemia causes oxidative stress include increased production of superoxide anion in the mitochondria [11], nonenzymatic glycation of proteins [12], and glucose autoxidation [13]. In addition, metabolic stress can result in changes in energy metabolism, reduced antioxidant defense, and increased levels of inflammatory mediators [14, 15].

Hyperlipidemia and altered antioxidant defenses are companions of oxidative stress. Diabetes-induced hyperlipidemia has been reported as one of the major risk factors for micro- and macrovascular complications [16]. Maintaining a balance between reactive oxygen species (ROS) and antioxidants is a major mechanism in preventing damage by oxidative stress; therefore, dietary supplementation of antioxidants could be a promising approach in the treatment of diabetes.

There is considerable interest in the potential beneficial effects of flavonoids on human health due to their biological activities which include antioxidant, antiviral, antiinflammatory, and antitumor activities [17]. Kolaviron (KV) is an extract from the bitter* kola* seeds (*Garcinia kola, *family:* Guttiferae) *containing a complex of* Garcinia* biflavonoids. KV has been proven to be beneficial in various pathological conditions of animal models through its antioxidative, antigenotoxic, analgesic, and anti-inflammatory properties [18–22], hence the need to explore its potentials in diabetic conditions. The present study investigated the beneficial effects of kolaviron on oxidative stress and inflammatory biomarkers in the blood of diabetic rats.

## 2. Materials and Methods

### 2.1. Chemicals

Streptozotocin (STZ), 6-hydroxydopamine, 6-hydroxy-2,5,7,8-tetramethylchroman-2-carboxylic acid (trolox) and 2-thiobarbituric acid (TBA), and *β*-nicotinamide adenine dinucleotide phosphate reduced tetrasodium salt (NADPH) were obtained from Sigma-Aldrich (Johannesburg, South Africa). Malondialdehyde bis (diethyl acetal) (MDA), hexane, and methanol were purchased from Merck (Johannesburg, South Africa). All other chemicals and reagents used were of the highest commercially available purity.

### 2.2. Animals

The study protocol was approved by the Faculty of Health and Wellness Sciences Research Ethics Committee of the Cape Peninsula University of Technology (Ethics Certificate number CPUT/HW-REC 2012/AO4). All the animals received humane care in accordance with the criteria outlined in the “Guide for the Care and Use of Laboratory Animals” prepared by the National Academy of Science (NAS) and published by the National Institute of Health (Publication number 80-23, revised 1978). Male Wistar rats (270 ± 25 g) were used for the study. Treatments were performed at the animal facility of the Medical Research Council (MRC), South Africa, and all standard operating procedures (SOPs) were strictly adhered to. All animals were housed individually at room temperature (22 ± 2°C) with 55 ± 5% humidity and an automatically controlled cycle of 12 h light and 12 h dark. Standard laboratory animal feed and water were provided* ad libitum *and animals were acclimatized to the experimental conditions for a period of one week before the commencement of the experiment.

### 2.3. Collection of Plant Material and Extract Preparation

*Garcinia kola* seeds were peeled, sliced, and air-dried (25–28°C). Kolaviron was isolated according to the method of Iwu et al. [23]. Briefly, the powdered seeds were extracted with light petroleum ether (bp 40–60°C) in a soxhlet for 24 hr. The defatted dried product was repacked and extracted with acetone. The extract was concentrated and diluted twice its volume with water and extracted with ethylacetate (6 × 300 mL). The concentrated ethylacetate yielded kolaviron, a golden yellow solid.

### 2.4. Experimental Design

Diabetes was induced in overnight fasted rats by a single intraperitoneal injection of a freshly prepared solution of streptozotocin (STZ; 50 mg kg^−1^ body weight) in citrate buffer (0.1 M, pH 4.5). Five days after STZ injection, diabetes was confirmed by a stable hyperglycemia (>18 mmol/L) in the tail blood glucose with a glucometer (Accu-Chek, Roche, Germany). The animals were divided into 4 groups (*n* = 10 per group): normal control (NC group), kolaviron treated normal control (KV), diabetic control (DM group), and kolaviron-treated diabetic group (DM + KV group). Kolaviron was dissolved in vehicle (dimethylsulphoxide (DMSO)) and administered orally at a dose of 100 mg kg^−1^ five times a week for six weeks. Normal control (NC) rats also received vehicle throughout the study period.

For biochemical estimations in the blood, rats were sacrificed under sodium pentobarbital anesthesia (60 mg/kg). Random blood glucose was determined in rats after collection of blood specimen from the abdominal aorta into glucose tubes. Blood samples were collected into tubes with or without EDTA to obtain plasma or serum, respectively, and centrifuged at 3500 g for 10 min at 4°C. Blood was also collected into another set of tubes used for HBA_1C_ estimation. Erythrocytes were obtained from EDTA-treated blood after plasma separation. Buffy-coat layers were discarded and erythrocytes were washed three times with cold saline and centrifuged at 3000 rpm for 10 min. Samples were haemolyzed by the addition of a threefold volume of ice-cold double distilled water (ddH_2_0) and the haemolysate was obtained after removing the cell debris by centrifugation at 3000 rpm for another 10 min. The supernatant was collected and stored at −80°C prior to the estimation of enzymatic activity.

### 2.5. Analysis of Glucose, Glycated Haemoglobin, and Lipid Profile

Plasma glucose, glycated haemoglobin (HBA_1C_), and serum lipid profile (total cholesterol, triglycerides) were analyzed with diagnostic kits in an automated clinical chemistry analyzer (Medical Cooperation, Bedford, MA, USA).

### 2.6. Plasma Antioxidant Capacity Assays

The antioxidant capacity of plasma samples was determined by the ferric-reducing antioxidant power (FRAP) assay of Benzie and Strain [24] with slight modifications in a Multiskan Spectrum plate reader (Thermo Fischer Scientific, Waltham, MA, USA). Oxygen radical absorbance capacity (ORAC) assay was conducted to kinetically measure the peroxyl radical scavenging activity in plasma samples with trolox as the antioxidant standard according to the method of Ou et al. [25]. The total plasma polyphenol was performed using the Folin Ciocalteu's phenol reagent according to the method of Singleton et al. [26].

### 2.7. Erythrocyte Antioxidant Enzyme Activity Assays

Activities of antioxidant enzymes in the erythrocytes were estimated in a clear 96-well plate using a Multiskan Spectrum plate reader (Thermo Fisher Scientific, USA). Catalase (CAT) activity was determined by the method of Aebi [27]. Superoxide dismutase was determined by the method of Crosti et al., [28], based on the inhibitory effect of SOD on the spontaneous autoxidation of 6-hydroxydopamine. Glutathione peroxidase (GPX) activity was determined according to the method of Ellerby and Bredesen [29] based on the oxidation of NADPH to NADP^+^ in the presence of H_2_O_2_. The protein concentrations of the erythrocyte were determined by the bicinchoninic acid (BCA) kit (Pierce, Illinois, USA).

### 2.8. Lipid Peroxidation (LPO)

Plasma malondialdehyde (MDA), an end product of lipid peroxidation, was determined by High-Performance Liquid Chromatography (HPLC) using a method adapted from Khoschsorur et al. [30]. Briefly, 100 *μ*L of plasma samples and standard MDA were mixed with 750 *μ*L orthophosphoric acid (0.44 M), 250 *μ*L of aqueous thiobarbituric acid (42 mM), and 450 *μ*L distilled water. The mixture was heated in a boiling water bath for 60 min. After cooling on ice, alkaline methanol (50 mL methanol + 4.5 mL 1 M NaOH) was added (1 : 1). The samples were centrifuged at 3500 g for 3 min at 4°C. 1 mL of supernatant was added to 500 *μ*L of n-hexane and the mixture centrifuged at 14000 g for 40 sec. 50 *μ*L of the supernatant was then chromatographed on an Agilent 1200 series HPLC. A 5 *μ*m YMC-PackPro C18 (150 mm × 4.6 mm i.d.) column was used for separation with 60 : 40 (v/v) 50 mM phosphate buffer (pH 6.8) and methanol, respectively, as mobile phase. The flow rate was 1 mL min^−1^. Fluorometric detection was performed with excitation at 532 nm and emission at 552 nm. The peak of the MDA-TBA adduct was calibrated with the MDA standard.

### 2.9. Assay of IL-1, MCP-1, and VEGF

The serum levels of inflammatory markers including monocyte chemotactic protein-1 (MCP-1), vascular endothelial growth factor (VEGF), and interleukin (IL)-1 were measured in the serum using Bio-Plex Pro magnetic bead-based assays (Bio-Rad Laboratories, Hercules, USA) on the Bio-Plex platform (Bio-Rad). Following previous optimization, samples were evaluated undiluted in a blinded manner. Samples were reacted with a mixture of fluorescent polystyrene beads bound with specific anticytokine primary antibodies, resulting in binding of the cytokines to the beads with the corresponding antibody. The biotinylated anticytokine secondary antibodies were then added and allowed to bind to the cytokine-bead complex followed by the addition of fluorescent phycoerythrin-conjugated streptavidin. All analytes levels in the quality control reagents of the kits were within the expected ranges. The standard curve for all the analytes ranged from 3 to 12000 pg/mL. Bio-Plex Manager software, version 6.0, was used for bead acquisition and analysis.

### 2.10. Statistical Analysis

Values were expressed as mean ± SD. Data were tested for normality and equality of variance using the Levene's test. Differences between groups mean were estimated using one-way analysis of variance (ANOVA) followed by the Student-Newman-Keuls test for all pairwise comparisons. The Kruskal-Wallis test, a nonparametric analogue to the one-way ANOVA, was used to test for group differences when data was not normally distributed. Result were considered statistically significant at *P* < 0.05 or marginally significant at *P* < 0.1. All the statistics were performed using MedCalc version 12.2.1 software (MedCalc software bvba, Mariakerke, Belgium).

## 3. Results

### 3.1. Kolaviron Treatment Lowered Blood Glucose, Glycated Haemoglobin (HBA_1C_), and Levels of Lipid Profiles

**Figure 1:**
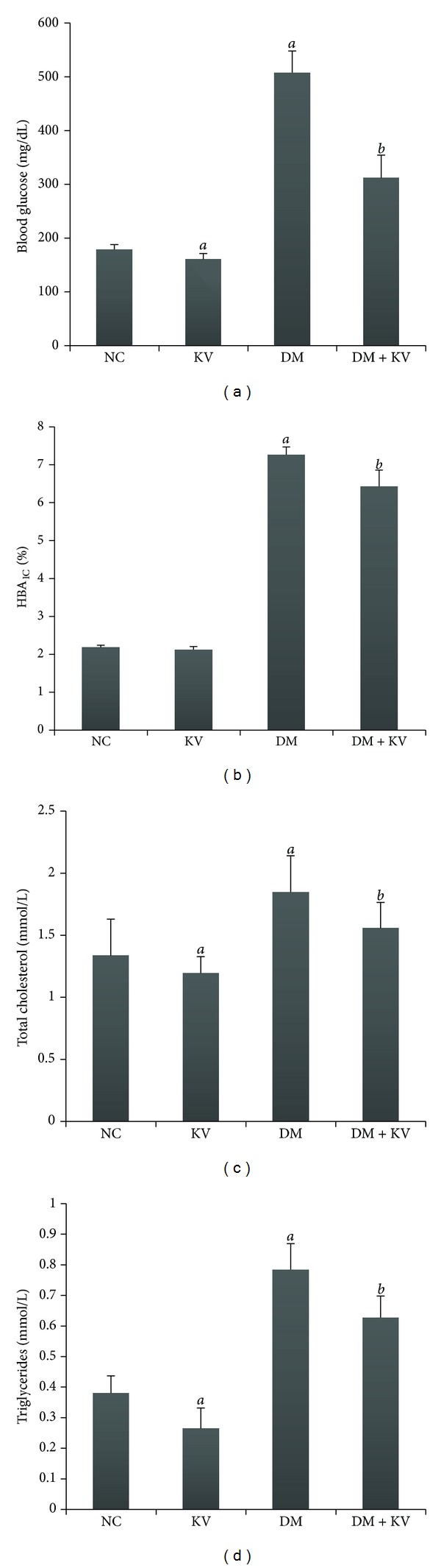
Effects of kolaviron on blood glucose, glycated haemoglobin, and levels of lipid profiles (total cholesterol and triglyceride concentrations). ^*a*^Values differ significantly from normal control (*P* < 0.05). ^*b*^Values differ significantly from diabetic control (*P* < 0.05). NC: normal control, KV: normal control treated with kolaviron, DM: untreated diabetic rats, DM + KV: diabetic rats treated with kolaviron.

Kolaviron treatment reduced total cholesterol and triglyceride concentrations in the serum of normal and diabetic rats as shown in Figure 1. Serum glucose concentration of the diabetic group was 2.84-fold higher than the nondiabetic group, indicating a sustained hyperglycemic state in the STZ-induced diabetic rats. Glycated hemoglobin (HBA_1C_) was significantly elevated in diabetic rats and the administration of kolaviron significantly lowered blood glucose and HBA_1C_ levels in diabetic rats.

### 3.2. Kolaviron Alleviates Oxidative Stress in the Erythrocyte of Diabetic Rats

A significant increase in GPX and SOD activities was observed in the erythrocytes of diabetic rats compared to control group (Table 1). This alteration was reversed after kolaviron administration to STZ-induced diabetic rats for 6 weeks. No significant change in CAT activity was observed. Diabetic rats showed increased level of the lipid peroxidation product, MDA. Kolaviron significantly reduced the formation of plasma MDA in STZ-diabetic model.

### 3.3. Effect of Kolaviron Treatment on Plasma Antioxidant Capacity

Although an increasing trend was observed in plasma antioxidant status assessed as FRAP, ORAC, and total polyphenols in diabetic rats, following kolaviron treatment for 6 weeks (Table 2), no statistically significant difference was observed in the estimated parameters in all treatment groups.

### 3.4. Kolaviron Abates Inflammation in the Serum of Diabetic Rats

Diabetic rats had significantly elevated serum levels of MCP-1, VEGF, and IL-1*β* compared to control rats (Table 3) and kolaviron normalized serum levels of these inflammatory markers in diabetic rats. No significant difference was observed in the serum levels of MCP-1, VEGF, and IL-1*β* in normal rats treated with kolaviron compared to untreated control group.

## 4. Discussion

The glucose lowering effect of kolaviron was observed in our study [31]. Glycated hemoglobin (HBA_1C_), expressed as a percentage of total blood hemoglobin concentration, is an effective index for the screening of glycemic control over time. Higher level of HBA_1C_ is observed in diabetes due to reaction of excess blood glucose with hemoglobin. Evidence of glycemic regulation by kolaviron is the significant reduction of blood glucose and glycated hemoglobin levels in kolaviron supplemented diabetic rats.

Increased glucose concentration results in oxidative stress. Erythrocytes are vulnerable to oxidative stress due to high concentration of polyunsaturated fatty acids, ferrous ions, and molecular oxygen [32]. Persistent hyperglycemia and increased oxidative stress are major players in the development of secondary diabetic complications such as nephrotoxicity [33] and hepatic injury [34, 35]. Cells maintain a variety of defenses against reactive oxygen species toxicity and oxidative stress. Among these are an array of antioxidant enzymes including superoxide dismutase (SOD), catalase (CAT), and glutathione peroxidase (GPX). Superoxide dismutase (SOD) scavenges superoxide radical by accelerating its conversion to hydrogen peroxide (H_2_O_2_) while glutathione peroxidase (GPX) detoxifies H_2_O_2_ and lipid peroxides [36, 37]. CAT acts in the decomposition of hydrogen peroxide (H_2_O_2_) to water and oxygen. Hyperglycemia can interfere with the antioxidant defense network and the alteration in the activity of antioxidant enzymes is a common occurrence in diabetes. However divergent results have been reported regarding the activities of antioxidants enzymes in diabetics.

Alteration in antioxidant defense in the diabetic rats was evidenced by a significant reduction in SOD activity in the erythrocyte of diabetic rats. The decrease in SOD activity in the hyperglycemic rats could be due to oxidative stress-induced inactivation. Increased H_2_O_2_ concentration, for example, is known to inactivate SOD [38]. Glycosylation of SOD and/or loss of Cu^2+^, a cofactor required for SOD activity, can also reduce SOD activity [39]. We observed that supplementation of kolaviron to diabetic rat increased SOD activity to near normal level.

The observed increase in erythrocyte GPX activity in the unsupplemented diabetic rats is an indication of increased H_2_O_2_ concentration. Increased GPX activity might be due to an increase generation of H_2_O_2_ and a compensatory response to erythrocyte membrane oxidative damage. The reduction of oxidative stress in diabetic rats by kolaviron is evidenced by the suppression of GPX activity. Some studies have reported an increase in erythrocyte catalase activity in diabetic rats [40] while others have shown a decrease [41] in erythrocyte CAT activity. However, similar results to ours were found by Bandeira and colleagues [42] who observed no significant difference in erythrocytes CAT activity. The erythrocyte protective effects of kolaviron against free radical attack may be due to its direct free radical scavenging capacity and improvement in antioxidant defense.

The release of free radicals during oxidative stress causes serious damage to the biological system by abstracting electrons from macromolecules causing instability and disintegration. Antioxidants, in general, are compounds and reactions which dispose, scavenge, and suppress the formation of ROS [43]. The ability of kolaviron to protect against free radical induced damage and lipid peroxidation process is also evidenced by the significant decrease in the levels of malondialdehyde (MDA)—a product of lipid peroxidation in the plasma of diabetic rats following treatment with kolaviron. Kolaviron contains* Garcinia* biflavonoids. Flavonoids are a diverse group of polyphenols (phenyl benzopyrans) which function as phytochemicals and are well known for their multidirectional biological activities [44]. Structure-activity relationships have been shown to play a major role in the antioxidant effects of flavonoids. Structural features which confer antioxidant and free radical scavenging activity on kolaviron include its multiple aromatic hydroxyl groups [45, 46].

The ORAC and FRAP assays are two antioxidant capacity assays commonly used to assess the total antioxidant capacity of biological samples [24, 25]. The ORAC assay relies on free radical damage to a fluorescent probe, most commonly fluorescein, caused by an oxidizing reagent resulting in a loss of fluorescent intensity over time. The inhibition of oxidative damage to the fluorescent probe can be correlated with the antioxidant capacity of the compound. Also, the FRAP assay measures the ability of a sample to reduce Fe^3+^ to Fe^2+^ and reflects the plasma levels of ascorbic acid, uric acid, and *α*-tocopherol [24] although it does not measure the SH-group-containing antioxidants. The antioxidant activity of a compound against a free radical does not necessarily match its reducing ability. No significant difference was observed in plasma antioxidant capacity assessed by FRAP and ORAC assay between all the groups in our study.

Lipids are main sources of peroxidation products and elevation in lipid levels in diabetes mellitus plays an important role in the development of atherosclerosis and represents an increased risk factor for coronary heart diseases [47]. The hyperlipidemia observed in the untreated diabetic rats in the present study could indicate an increase in the mobilization of free fatty acids from the peripheral fat depots. This could result from the uninhibited actions of lipolytic enzyme lipase caused by insulin deficiency characteristic of the diabetic state. We observed in the present study that kolaviron significantly lowered total cholesterol and triglyceride levels in the serum of normal and diabetic rats.

Hyperglycaemia can result in elevated levels of circulating inflammatory mediators [48]. Increase in oxidative stress can increase the production of inflammatory proteins and vice versa. Interaction between oxidative stress and inflammatory signals plays a major role in disease progression and tissue damage in diabetes [49, 50]. Elevated levels of VEGF, IL-1, and MCP-1 have been implicated in diabetic-related complications [51–53]. VEGF is an angiogenic factor with potent vascular permeability and angiogenic effects, plays a central role in vasculogenesis and neoangiogenesis by promoting the survival, migration, and proliferation of endothelial cells, and regulates glomerular permeability [54]. Our findings agree with previous report of elevated serum levels of VEGF in diabetes [55]. Despite its protective role in nondiabetic renal disease, overexpression of VEGF is implicated in diabetic renal disease by increasing the permeability of vascular endothelium, endothelial cell proliferation and migration, and activation of matrix-degrading and plays a major pathophysiological role in diabetic nephropathy. Factors that modulate expression of VEGF and its receptors are high glucose, AGEs, endothelin 1, angiotensin II, and TGF-*β* [56]. In the present study, kolaviron normalized VEGF concentration in the serum of diabetic rats. The role of IL-1*β* has been well reported in diabetes. IL-1*β* increases the expression of chemotactic factors and adhesion molecules, enhances vascular endothelial permeability, and stimulates the proliferation of mesangial cells and matrix synthesis [49]. Kolaviron significantly reduced IL-1*β* mediated inflammation in the serum of diabetic rats in comparison to untreated diabetic control rats.

Increased production of monocyte chemoattractant protein (MCP-1) can occur after induction of oxidative stress or growth factors in a number of cells such as monocytes, smooth muscle cells, and endothelial cells [57]. MCP-1 mediated macrophage accumulation plays a role in the formation of vascular inflammation, atherosclerotic lesion, and kidney damage [58–60]. The protective effect of kolaviron in our study is evidenced by the restoration of MCP-1 to near normal levels in diabetic rats.

Overall, our study showed the protective effects of kolaviron in the blood of diabetic rats through improvement in glucose level, endogenous antioxidant defense, and inflammation. Increased activation of major pathways such as advanced glycation end products (AGEs), protein kinase C (PKC), and aldose reductase due to a hyperglycemic state is thought to amplify the production of free radicals and inflammatory biomarkers in diabetes, hence mediating the damaging effect. The ability of kolaviron to interfere with one or more of these pathways in the present study could possibly explain the observed beneficial effects of kolaviron in the blood of diabetic rats.

## 5. Conclusion

The result of this study indicates that the mechanism of antidiabetic effects of kolaviron may be related to its intrinsic antioxidative and anti-inflammatory properties and suggests that kolaviron may be beneficial in reducing the risk of vascular complications in diabetes.

## Figures and Tables

**Table 1:** Effects of kolaviron on erythrocyte enzymatic activities and plasma lipid peroxidation in diabetic and normoglycemic rats.

	CAT	SOD	GPX	MDA
NC	0.34 ± 0.06	0.029 ± 0.008	32.13 ± 4.60	1.60 ± 0.20
KV	0.28 ± 0.12	0.026 ± 0.008	35.29 ± 4.14	1.5 ± 0.21
DM	0.37 ± 0.07	0.018 ± 0.004^a^	48.30 ± 8.21^a^	2.01 ± 0.3^a^
DM + KV	0.36 ± 0.11	0.022 ± 0.006	30.60 ± 8.43^b^	1.61 ± 0.31^b^

Table 1: Illustrates the effect of kolaviron on erythrocyte enzymatic activities and plasma lipid peroxidation in diabetic and normoglycemic rats. CAT: catalase, *μ*mol H_2_O_2_ consumed/min/mg protein, SOD: superoxide dismutase, U/*μ*g protein, GPX: glutathione peroxidase, *μ*mol NADPH oxidized/min/*μ*g protein, MDA: malondialdehyde, (*μ*mol MDA/L). Data are presented as mean ± S.D. ^a^Values differ significantly from normal control (*P* < 0.05). ^b^Values differ significantly from diabetic control (*P* < 0.05). NC: normal control; KV: normal control treated with kolaviron; DM: untreated diabetic rats; DM + KV: diabetic rats treated with kolaviron.

**Table 2:** Effects of kolaviron supplementation on plasma antioxidant status in diabetic rats.

	ORAC (*μ*mol TE/L)	FRAP (*μ*mol AAE/L)	Total polyphenol (mg GAE/L)
NC	11.72 ± 1.27	85.68 ± 9.45	48.39 ± 4.5
KV	13.57 ± 1.54	82.87 ± 9.26	47.84 ± 3.3
DM	11.20 ± 1.0	80.60 ± 8.56	43.73 ± 5
DM + KV	11.03 ± 1.71	92.98 ± 15.95	46.45 ± 6.15

Data as shown in Table 2 are presented as mean ± S.D. NC: normal control; KV: normal control treated with kolaviron; DM: untreated diabetic rats; DM + KV: diabetic rats treated with kolaviron. AAE: ascorbic acid equivalent; TE: trolox equivalent; FRAP: ferric-reducing ability of the plasma; ORAC: oxygen radical absorbance capacity.

**Table 3:** Effects of kolaviron treatment on levels of inflammatory proteins and growth factor.

	IL-1*β* (pg/mL)	MCP-1 (pg/mL)	VEGF (pg/mL)
NC	28.37 ± 10.54	226.62 ± 75.71	10.45 ± 1.4
KV	20.48 ± 5.13	231.26 ± 78.31	9.49 ± 2.5
DM	43.32 ± 8.65^a^	542.77 ± 67.27^a^	15.41 ± 1.96^a^
DM + KV	16.43 ± 7.74^b^	266.61 ± 81.36^b^	12.44 ± 1.73^b^

Table 3 shows effects of kolaviron on interleukin (IL)-1*β*, monocyte chemotactic protein (MCP-1), and vascular endothelial growth factor (VEGF). Data are presented as mean ± S.D. ^a^Values differ significantly from normal control (*P* < 0.05). ^b^Values differ significantly from diabetic control (*P* < 0.05). NC: normal control; KV: normal control treated with kolaviron; DM: untreated diabetic rats; DM + KV: diabetic rats treated with kolaviron.
